# Chronic complications of type 2 diabetes and associated factors: a cross-sectional study at the Moulay Hassan Hospital in Kenitra, Morocco

**DOI:** 10.11604/pamj.2024.49.84.42930

**Published:** 2024-11-21

**Authors:** Mohammed El aameri, Imane Jaghror, Nadia Meskini, Hiba Benchehida, Ibtissam Eladha, Miloud Chakit, Aroui Norelhoda, Bouchra Taib, Youness Taboz

**Affiliations:** 1 Faculty of Sciences, Natural Resources and Sustainable Development Laboratory, Ibn Tofail University, Kenitra, Morocco,; 2 Biology and Health Laboratory, Ibn Tofail University, Sciences Faculty, Kenitra, Morocco,; 3 Department of Biology and Health, Cognitive-Behavioral Neurosciences and Applied Nutrition Unit, Kenitra, Morocco,; 4 Department of Biology, Faculty of Science, Ibn Tofail University, Kenitra, Morocco

**Keywords:** Type 2 diabetes, complications, cardiovascular diseases, hygienic-dietary, Morocco

## Abstract

**Introduction:**

all around the world, type 2 diabetes is considered a metabolic disease that generates complications that can be very serious, even fatal, over time, especially if not properly managed. Clinical and biological parameters in blood glucose levels will be assessed in this study (N=300 patients). We assess the prevalence of complications caused by diabetes including diabetic retinopathy, nephropathy, neuropathy, and cardiovascular diseases. Type 2 diabetes is an incurred disease, but it can be managed. Self-therapeutic education is therefore imperative and highly recommended.

**Methods:**

this study was carried out at the Moulay Hassan Hospital in Kenitra (Morocco) using a self-administered questionnaire targeting chronic complications caused by diabetes. Descriptive statistical analysis was followed to determine frequencies and percentages for complications and we carried out univariable and multivariable regression analysis to determine factors associated with complications.

**Results:**

the study highlights clinical and bioclinical features. Mean age of patients (58.51±13.11 year with standard deviation: 13,113), hypertension (45.7%), glycemia (1.85±0.64 g/l), HbA1c (8.09±1.7%), BMI (26.44±3.4 kg/m^2^) and chronic complications (41.7%) including retinopathy (16%), nephropathy (4%), neuropathy (3.3%), cardiovascular diseases (16.7%) and amnesia (2%). There was mainly a statistical difference between complications and HTA: (AOR=2.43 (1.52-3.89) (CI=95%) (p=0.000), chronic complications and smoking: (AOR=0.16) (0.04-0.61) (CI=95%) (p=0.007), complications and physical activity: (AOR=3,34) (1.34-7.24) (CI=95%) (p=0.014) and complications and lipid profile: (AOR=4.95) (2.79-8.77) (CI=95%) (p=0.001).

**Conclusion:**

therapeutic education of type 2 patients with diabetes remains highly recommended, as it improves compliance with non-pharmacological treatment, especially hygienic-dietary measures, and physical activity, and limits the early onset of complications associated with diabetes.

## Introduction

Type 2 diabetes mellitus (T2DM), affecting approximately 463 million people in 2019, and projected to rise to 700 million by 2045 is a global public health concern-particularly in low-income and middle-income countries. This exponential increase in diabetes incidence has far-reaching implications for public health systems, economies, and, most importantly, the lives of individuals affected. A conglomeration of modifiable risk factors including physical inactivity, unhealthy dietary patterns, increased blood pressure, dyslipidemia, and obesity, alongside nonmodifiable risk factors such as an aging population, family history of diabetes, and ethnicity are driving the current rise of T2DM and its complications in Africa. Microvascular complications, comprising nephropathy, neuropathy, and retinopathy, along with macrovascular complications, including coronary artery disease, peripheral artery disease, and cerebrovascular disease, are the major cause of mortality and morbidity in patients with type 2 diabetes (T2DM patients) [1]. Clinical complications in untreated or poorly controlled type 2 diabetes are responsible for significant morbidity and mortality: being diabetic increases the risk of death, particularly from cardiovascular causes, compared with the general non-diabetic population [2]. Chronic complications include macroangiopathy, defined as damage to medium- and large-caliber arteries. This includes damage to coronary arteries, arteries destined for the neck, and those of the lower limbs. Cardiovascular complications are a major problem for type 2 diabetic subjects since nearly three-quarters of them die of cardiovascular causes [3].

Several epidemiological studies have shown that the incidence of microvascular complications is associated with more severe glycemic imbalance. This was demonstrated in particular by the United Kingdom Prospective Diabetes Study (UKPDS), which clearly indicated that the mean HbA1c (glycemic hemoglobin) level over an 11-year follow-up was associated with a higher incidence of retinopathy, nephropathy, and neuropathy [4]. Therapeutic targets in the prevention of diabetic complications are threefold: glycemic, blood pressure, and lipid. Chronic complications of diabetes occur on average after 10 years of poorly controlled diabetes. Their insidious nature and lack of symptoms usually explain their late diagnosis. They are a source of morbidity, mortality, blindness, and disability. They must be prevented by proper management of diabetic patients because once they have set in, they are irreversible [5]. The association between vascular risk and even a moderate rise in blood sugar levels has been demonstrated, but correct blood sugar control alone cannot prevent this risk. The combination of hygienic and dietetic rules, including the fight against a sedentary lifestyle and excess weight, the cessation of smoking, and the prescription of multi-drug therapy including, in particular, the following can significantly reduce the risk of cardiovascular events and death [6]. The aim of our study is to determine the prevalence of chronic complications caused by T2DM, and also to study statistically the existing relationships between these complications and other bioclinical parameters.

## Methods

**Study design and setting:** according to the census of 2014, the number of inhabitants of the city of Kenitra is estimated at 1034114. The number of hospital establishments in Morocco is 170 (2024). The regional and prefectural hospital centers are 17. According to the Ministry of Health, the number of people with diabetes in Morocco is estimated at 3 million. This was a descriptive, cross-sectional observational study conducted over 6 months in a public hospital (Moulay Hassan) (Diabetology Department) in Kenitra Province (Morocco). On an affective of 300 T2DM patients. The survey took place at the diabetes association, which helps diabetic patients whose socio-economic situation is relatively weak.

**Study population:** the size of the sample was limited to 300 patients with diabetes who came to the hospital to have their blood glucose and HbA1c levels checked. This was a cross-sectional study conducted at a public hospital. This public hospital helps diabetic patients, especially those whose financial situation is precarious. All the clinical and biological variables related to glycemia have been determined: glycemia, weight, height, body mass index (BMI), blood pressure (BP), HbA1c, diabetes control, diabetes monitoring method, type of medication, level of sporting activity, and genetic link. Glycemic hemoglobin, genetic link, hypoglycemia, glycemic control, type of treatment (insulin and oral antidiabetics), diabetes monitoring, and duration of diabetes on chronic complications. All T2DM patients with glycemia levels strictly above 1,26 g/l were included. Type 1 diabetic patients are excluded from the study, as well as gestational diabetes.

**Definitions:** diabetic complications are confirmed by a physician if patients exhibit one or more chronic issues like neuropathy, nephropathy, retinopathy, impotence, peripheral sensory pains or diabetic foot ulcers. Other clinical and biological parameters must be assessed to better study and recognize the source of complications: BMI, blood glucose, physical activity, BP, and duration of diabetes.

**Statistical study:** statistical analysis was carried out using IBM SPSS 25 statistical analysis software. After coding the data, descriptive analyses were listed to meet the objectives and study diabetes as a function of the different variables. Bivariate analyses were chosen to study the relationships between the different variables. Chi-square tests were performed for quantitative variables. The adjusted odds ratios and 95 % confidence intervals were obtained at a level of significance of 5%. We carry out univariable and multivariable regression analyses to determine factors associated with complications in T2DM patients.

**Data collection:** in addition to the determination of chronic diseases, the biological parameters have been determined. The equipment used consists mainly of a blood pressure monitor, a weighing scale, a ruler, blood glucose strips, a hemoglobinometer, and a questionnaire designed especially for diabetic people. In addition to the clinical and biological variables. Blood pressure, height, weight, blood glucose, and glycemic HbA1c are measured on the spot. We have found it difficult to measure waist circumference, which is also an essential parameter, given the refusal of most rural women to do so. Despite the COVID-19 crisis, a great deal of effort has gone into this work. The association that helps diabetic people has done us a great service by offering favorable conditions for carrying out this medical research.

**Laboratory analysis:** biological medical tests are carried out in the hospital's on-site laboratory, usually on an empty stomach, to check blood sugar levels and HbA1c. For some T2DM patients with serious complications, other biological tests are required namely the lipid profile (cholesterol and triglycerides) and the renal profile (creatinine and albuminuria).

**Ethical considerations:** our study complies fully with the ethical recommendations for medical research in Morocco. The questionnaire is characterized by absolute anonymity. The research was carried out with the agreement of the dean of the Kenitra Faculty of Science and the president of the association. Our research follows the principles of the World Medical Association's (WMA) declaration of Helsinki, which is a statement of ethical principles for medical research involving human subjects.

## Results

**Socio-demographic characteristics:** the study population comprised women 162 (54%) and men 138 (46%), with a sex ratio of 0.85. The mean age of diabetic people was (58.51 ± 13.11) years with a standard deviation: of 13.113. The age-class distribution showed that the age group between 40 and 60 was the most dominant with 141 (46.9%). Regarding the professional situation of T2DM patients, 43 (14.3%) of them are civil servants, 85 (28.3%) work in the private sector and 66 (22%) are unemployed 106 (35.3%) of the patients work on a daily or permanent basis. In addition, 173 (57.7%) of T2DM patients are married, 73 (24.3%) are widowed, 42 (14%) are divorced and 21 (7%) are single. The educational level of T2DM patients is unstable. In fact, 51 (17%) have a primary school education. Women account for a large percentage of illiterate T2DM patients 44 (14.6%), 48 (16%) have secondary education, 81 (27%) have high school education and 39 (13%) in university education. T2DM patients live in both urban 246 (82.2%) and rural 54 (17.7%) areas.

**Clinical and biological characteristics:** the results for BMI are as follows: the T2DM patients with a BMI of less than 26 are represented by 137 (45.6%), the T2DM with a BMI of (26-30) (overweight) are 131 (43.6%) and the T2DM patients with a BMI of more than 30 (obese) are 32 (10.6%). The percentage T2DM whose BP remains normal is 163 (54.3%), while 137 (45.7%) have abnormal BP (≥13 mmHg). Women are more hypertensive than men, with 82 (27.3%) versus 55 (18.3%). In our population, the mean blood glucose level was (1.85±0.64 g/l), with a minimum value of 0.56 g/l and a maximum value of 4.56 g/. The group with the highest glycemia is [1.^26^-^2^] with 44 (53.3%), followed by the (2-3) group with 76 (25.3%), and T2DM patients with glycemia lower than 1.26 g/l with 44 (14.7%). The T2DM patients with glycemia higher than 3 with 20 (6.7%). The biological mean HbA1c is 8.09±1.7%. The minimum value is 4.5%, while the maximum is 14.2%. T2DM patients with an HbA1c of less than 8 % account for 169 (56.3%), while T2DM patients with an HbA1c of more than 8% account for 131 (43.7%). Women´s blood glucose and hbA1c percentages are higher than men's. Regarding treatment regularity, 225 (75%) of T2DM patients claim to follow a regular treatment, compared with 75 (25%) who do not follow a regular treatment (Table 1). Young patients (<40 years) showed lower rates of complications and risk factors, indicating the potential benefits of early intervention and lifestyle modifications.

**Table 1:** socio-demographic and biologic characteristics of patients with diabetes (N=300)

Variables	Frequency	Percentage (%)
**Age**		
< 40	28	9.3
[41-70]	216	72
>70	56	18.7
**Gender**		
Women	162	54
Men	138	46
**BMI (Kg/M^2^)**		
< 26	137	45.6
[26-30]	131	43.6
>30	32	10.6
**BP (mmHg)**		
Yes	163	54.3
No	137	45.7
**Hypoglycemia**		
Yes	104	34.7
No	116	65.3
**Genetic link**		
Yes	191	63.7
No	109	36.7
**Physical activity**		
Yes	169	56.3
No	131	43.7
**Regularity of treatment**		
Yes	226	75.3
No	74	24.7
**HbA1c (%)**		
< 8	169	56.3
> 8	131	43.7
**Complications**		
Yes	125	41.7
No	175	58.3

**Prevalence of complications:** in our study, the prevalence of complications associated with diabetes gave negative results with 175 (58.3%) and positive results with 125 (41.7%). Women [93 (31%)] are more affected by complications than men [82 (27.3%)]. Some T2DM patients have one or more complications at the same time. The age group most affected by complications is [51-60] with 55 (18.3%), followed by the (41-50) group with 41 (14%). In terms of degenerative complications, retinopathy accounts for 48 (16%), nephropathy for 12 (4%), neuropathy for 10 (3.3%), cardiovascular disease for 50 (16.7%), amnesia for 5 (1.7%), and 175 (58.3%) have no complications at all. The (61-70) age group has the highest percentage of retinopathy 19 (6.3%), nephropathy 4 (1.3%) and neuropathy 4 (1.3%), while the highest percentage of cardiovascular disease is found in the [51-60] age group 14 (4.6%), followed by the (61-70) age group 12 (4%) (Table 2).

**Prevalence of complications:** in our study, the prevalence of complications associated with diabetes gave negative results with 175 (58.3%) and positive results with 125 (41.7%). Women [93 (31%)] are more affected by complications than men [82 (27.3%)]. Some T2DM patients have one or more complications at the same time. The age group most affected by complications is [51-60] with 55 (18.3%), followed by the (41-50) group with 41 (14%). In terms of degenerative complications, retinopathy accounts for 48 (16%), nephropathy for 12 (4%), neuropathy for 10 (3.3%), cardiovascular disease for 50 (16.7%), amnesia for 5 (1.7%), and 175 (58.3%) have no complications at all. The (61-70) age group has the highest percentage of retinopathy 19 (6.3%), nephropathy 4 (1.3%) and neuropathy 4 (1.3%), while the highest percentage of cardiovascular disease is found in the [51-60] age group 14 (4.6%), followed by the (61-70) age group 12 (4%) (Table 2).

**Table 2:** distribution of complications associated with diabetes and cardiovascular risk factors according to gender and age of type 2 diabetes mellitus patients

Complications (N=300)	Gender		Age		
	Men	Women	< 40	[41-70]	>70
	F (%)	F (%)	F (%)	F (%)	F (%)
None	81 (27)	94 (31.3)	20 (6.6)	129 (43)	24 (8)
**Retinopathy**	18 (6)	30 (10)	2 (0.7)	37 (12.3)	8 (2.6)
**Nephropathy**	6 (2)	6 (2)	0	8 (2.6)	4 (1.3)
**Neuropathy**	6 (2)	4 (1.3)	1 (0.3)	7 (2.2)	2 (0.6)
**Cardiov. diseases**	23 (7.7)	27 (9)	2 (0.7)	31 (10.4)	17 (5.6)
**Cardiovascular risk factors**					
**No risk factors**	81 (27)	114 (38)	25 (8.3)	13 (45.9)	31 (10.3)
**Smoking**	48 (16)	3 (1)	2 (0.7)	37 (12.3)	12 (4)
**Alcoholism**	6 (2)	0	0	3 (0.9)	3 (1)
**Obesity**	12 (4)	21 (7)	1 (0.3)	26 (8.6)	6 (2)
**Inactivity**	5 (1.7)	10 (3.3)	0	12 (3.9)	3 (1)

**Factors associated with complications:** our study focuses on the main factors that can promote chronic complications in T2DM patients, in particular age, gender, BP, genetic link, BMI, glycemia, HbA1c, lipid profile, physical activity, cigarette smoking, and regularity of treatment. Our research shows a highly significant relationship between complications and the age of T2DM, especially in the age groups [41-50] with AOR: 4.889 (1.717-13.921) (CI=95%) (p=0.003) (p<0.05); [51-60]: AOR: 3.216 (1.482-6.976) (CI = 95%) (p=0.003) (p<0.05); [61-70]: AOR: 2.619 (1.303-5.263) (CI=95%) (p=0.007) (p<0.05). Patients aged [41-70] years, had an increased risk of complications. There is no significant increase in risk for patients over 70 years. There was also a very significant relationship between complications and patients' BP, with AOR: 2.435 (1.521-3.897) (CI =95%) (p=0.000) (p<0.05). There was also a very strong significant relationship between chronic complications and the factor (lipid profile) with AOR: 4.954 (2.798-8.770) (CI=95%) (p=0.001) (p<0.05). Same finding between chronic complication and physic activity: AOR: 3.345 (1.342-7.245) (CI=95%) (p=0.014) (p<0.05). The existence of an important statistical link between complications and cigarette smoking has also been revealed with AOR: 0.166 (0.045-0.613) (CI=95%) (p=0.007) (p<0.05). On the other hand, our study revealed no statistically significant link between chronic complications and glycemia: [1.^26^-^2^] g/l with AOR: 1.31 (0.45-3.79) (CI=95%) (p=0.612), [-3] g/l with AOR: 1.50 (0.39-3.80) (CI=95%) (p=0.324) and glycemia > 3 g/l with AOR: 1.37(0.51-3.69) (CI=95%) (p=0.527), nor complications and treatment regularity with AOR: 1.06 (0.75-1.86) (CI=95%) (p=0.556) and nor complications and BMI: [26-30] Kg/M^2^ with AOR: 1.37 (0.79-3.77) (CI=95%) (p=0.166), and BMI > 30 Kg/M^2^ with AOR: 1.52 (0.82-3.6) (CI=95%) (p=0.134) (Table 3). According to our research, factors such as age, BP, physical activity, lipid profile and smoking appear to be predictive of the occurrence of complications. While glycemia, BMI, and regularity of treatment do not generate any complications. Complications associated with diabetes in T2DM patients are mainly influenced by age, the presence of hypertension and lipid problems, while physical activity is a protective factor.

**Table 3:** distribution of complications associated with diabetes according to different bioclinical parameters of type 2 diabetes mellitus patients

Variables		Diabetes-related complications			
	F (%)	CORs (CI= 95%)	P-value	AORs (CI= 95%)	P-value
**Age**					
< 40	28 (9%)	Ref		Ref	
[41-50]	58 (19%)	4.76 (1.82-13.86)	0.003	4.88 (1.71-13.92)	0.003
[51-60]	83 (28 %)	3.36 (1.52-7.03)	0.003	3.21 (1.48-6.97)	0.003
[61-70]	75 (25 %)	2.68 (1.36-6.36)	0.007	2.61 (1.30-5.26)	0.007
>70	56 (19 %)	1.15 (0.58-2.15)	0.914	1.04 (0.52-2.10)	0.896
**Gender**					
Men	162 (54%)	1.13 (0.71-1.78)	0.822	1.08 (0.68-1.71)	0.725
Women	138 (46%)	Ref		Ref	
**Body mass index (Kg/m^2^**					
< 26	137 (46%)	Ref		Ref	
[26-30]	131 (43%)	1.85 (0.86-3.96)	0.110	1.73 (0.79-3.77)	0.166
>30	32 (11%)	1.58 (0.94-3.91)	0.142	1.52 (0.82-3.67)	0.134
**Glycemia (g/l)**					
< 1,26	44 (15 %)	Ref		Ref	
[1.26-2]	160 (53 %)	1.39 (0.50-3.86)	0.670	1.31 (0.45-3.79)	0.612
[2-3]	76 (25 %)	1.56 (0.64-4.86)	0.415	1.50 (0.59-3.80)	0.394
> 3	20 (7 %)	1.42 (0.56-3.74)	0.550	1.37 (0.51-3.69)	0.527
**Arterial hypertension (mm Hg)**					
Yes	163 (54.3 %)	2.48 (1.48-3.75)	0.000	2,43 (1.52-3.89)	0.000
No	137 (45.7%)	Ref		Ref	
**Lipid profile**					
Yes	74 (25%)	5.15 (2.88 -8.89)	0.001	4.95 (2.79-8.77)	0.001
No	226 (75%)	Ref		Ref	
**Physical activity**					
Yes	169 (56.3%)	3.42 (1.38-7.27)	0.02	3.34 (1.34-7.24)	0.014
No	131 (43.7%)	Ref		Ref	
**Genetic link**					
Yes	191 (63.7%)	Ref		Ref	
No	109 (36.3 %)	1.35 (0.89-1.98)	0.572	1.15 (0.75-1.86)	0.556
**Smoking**					
Yes	51 (17%)	0.21 (0.08-0.75)	0.004	0.16 (0.04-0.61)	0.007
No	249 (83%)	Ref		Ref	
**Regularity of treatment**					
Yes	226 (75%)	1.23 (0.75-1.93)	0.846	1.06 (0.62-1.81)	0.821
No	74 (25%)	Ref		Ref	

## Discussion

**Figure 1:**
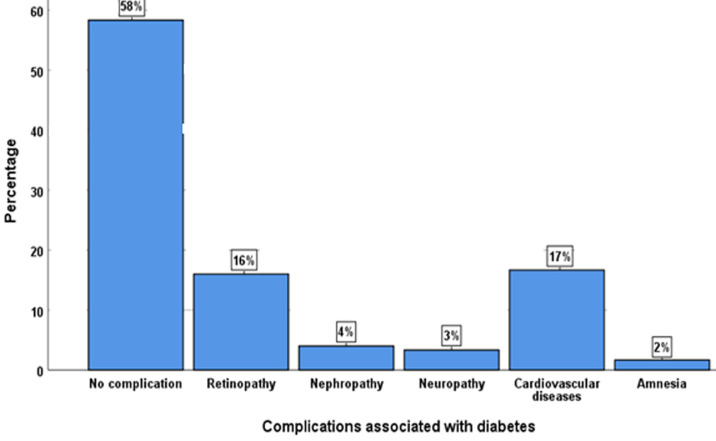
distribution of chronic complications in type 2 patients with diabetes

The main objective of our medical study is to assess the prevalence of chronic complications caused by diabetes and also to determine any significant links between these chronic complications and certain clinical and biological factors. In our case, BMI, gender, genetic link, and glycemia account for diabetes complications. Our research shows that 174 (58%) of T2DM patients have no chronic complications caused by diabetes, while 126 (42%) have complications as follows: retinopathy 48 (16%), regularity of treatment BMI are not statistically significant in predicting complications associated with diabetes. While factors such as age, arterial hypertension (HTA), lipid profile and physical activity are statistically significant nephropathy 12 (4%), neuropathy 12 (3%), cardiovascular disease 51 (17%) and amnesia 6 (2%) (Figure 1). Cardiovascular diseases account for the largest percentage (17%). In our study, most T2DM patients with diabetes-related complications had diabetes for more or less a long time (10 years or more). In some cases, T2DM patients are even unaware of their condition, since in some cases diabetes is silent (asymptomatic), prolonging the course of the disease and worsening the diabetic patient's health. The HbA1c observed in T2DM patients generally remains high, representing complications (7.5%). The measurement of these parameters enables adequate follow-up during the medical management of diabetes. However, it is also necessary to monitor other cardiovascular risk factors. A UKPDS study shows that lowering BP in T2DM, combined with glycemic control, significantly reduces the risk of macroangiopathic and microangiopathic complications. The appearance of complications depends on the duration of diabetes and the intensity of hyperglycemia. Arterial hypertension and, more generally, cardiovascular risk factors (overweight, increased blood lipid levels, smoking) can also be factors in the aggravation of microangiopathies [7]. Age, HTA, and lipid profile are strong predictors of diabetes-related complications.

Physical activity is associated with reduced risk, highlighting its importance in managing T2DM. Interestingly, smoking appears to correlate with lower odds of complications, which may require further investigation into confounding factors or biases. Other parameters like gender, BMI, glycemia levels, genetic links, and treatment regularity do not show significant associations in this dataset. This analysis emphasizes the need for targeted interventions based on age, hypertension management, and lifestyle modifications, particularly physical activity. Good control of HbA1c, treatment of hypertension, and the implementation of hygienic-dietary measures will limit the risk of microangiopathies and delay their aggravation. However, there is no HbA1c threshold below which there is no risk of diabetic complications [8]. Cardiovascular disease (17% in our case) is considered the most lethal pathology of T2DM [9]. Patients with poorly controlled diabetes frequently develop micro and macrovascular complications and evidence from large controlled clinical studies suggests that intensive glycemic control can significantly reduce their risk of development and/or progression [10]. Until recently, the predominant focus of diabetes treatment has been on lowering HbA1c levels. However, control of fasting hyperglycemia alone is insufficient to obtain optimal glycemic control as evidence suggests that reducing postprandial plasma excursion is as important, or perhaps more important for achieving desired glycemic targets [11]. Diabetic retinopathy remains silent for many years. It only becomes symptomatic when complications arise. In our case, retinopathy accounted for 84 (16%), with only one case of blindness [12].

Nephron capital is currently considered a major risk factor for the development of hypertension as well as epigenetic and environmental factors, dietetics [13,14], lifestyle, and physical activity [15]. is the leading cause of chronic kidney disease in industrialized countries. In Morocco, its prevalence according to the National Population and Family Health Survey is 3.3% [16]. The risk of renal failure increases with age and hypertension. Diabetic nephropathy is the leading cause of end-stage renal failure in most Western countries. Around 15% of T2DM patients develop kidney failure after 10 to 25 years [17]. Microangiopathy complications associated with diabetes include diabetic retinopathy, which progresses over 15 to 20 years to reduced visual acuity and even blindness; diabetic nephropathy, which progresses to persistent proteinuria and then end-stage renal failure. Peripheral neuropathy, which is initially subclinical, then gives rise to distal symptoms, predominantly in the lower limbs (sensory-motor disorders) [18]. According to the World Health Organization (WHO), the risk of amputation is ten times higher in diabetics. In our study, despite a limited sample size (N=300), we found that 47% of T2DM patients were hypertensive and generally elderly. Environmental or genetic factors, such as a sedentary lifestyle, high BMI, or high body weight, aggravate BP and further promote the onset of related complications in type 2 diabetic patients. Hypertension frequently precedes the discovery of diabetes. In cases of microalbuminuria, hypertension is responsible for kidney damage in only a third of cases [19,20]. However, type 2 diabetes is frequently part of a metabolic syndrome, characterized by hypertension, diabetes or glucose intolerance, obesity, hypertriglyceridemia, and hypo-high density lipoprotein (HDL) anemia [21].

The metabolic syndrome is attributed to insulin resistance, which is thought to be involved in the pathophysiology of essential hypertension [22]. Hypertension is common in diabetic patients and carries a high risk of cardiovascular disease and nephropathy. Its treatment is part of a comprehensive approach to diabetic pathology, including weight control, especially in T2DM [23]. A correlation between glycemic control as measured by HbA1c and the degenerative complications of T2DM patients has been established by large-scale epidemiological studies such as the Diabetes Control and Complications Trial (DCCT), (UKPDS). Good glycemic control helps reduce the costs of early hospitalization and retirement due to established complications. But to achieve these objectives, it is necessary to combine these biological monitoring measures with patient awareness and education programs to ensure good compliance [24]. The results of several prospective epidemiological studies indicate that regular physical activity reduces the risk of developing type 2 diabetes in adulthood in the general population and in glucose-intolerant subjects. In addition to the preventive role of physical activity, there is a body of data demonstrating its importance in the therapeutic plan for patients with T2DM. Physical activity and diet are the pillars of management. If practiced regularly and with moderate intensity, physical activity can help reduce the risk of complications associated with T2DM by 30-50%. Regular physical activity reduces insulin resistance in non-insulin-dependent diabetics. Exercise leads to: improved insulin sensitivity, lower blood sugar levels, reduced cardiovascular risk thanks to its favorable effect on BP, lipid balance and weight control, as well as physical and psychological well-being [25,26].

**Limitation of the study:** the study's limitations lie in the fact that we were unable to measure waist and hip circumferences due to women's customs. We would have counted more patients with diabetes if the covid-19 pandemic had not existed. Some diabetic subjects came to have their glycemia checked without HbA1c. In this case, we noted the last medical analysis of HbA1c, which was no more than three months old.

## Conclusion

Diabetes is an endocrine disease that cannot be cured but can be controlled. The evaluation of chronic complications in diabetic subjects has revealed worrying percentages that worsen the health status of patients. The results are subsequently increased morbidity and mortality among T2DM patients. Patients are seriously lacking in awareness about diabetes. Most patients are unaware of the importance of physical activity in the management of diabetes and are not in a position to provide primary care as early as possible in order to limit as far as possible the complications caused by diabetes. Hygienic and dietary measures are the first strategies to be followed before taking medication. Awareness-raising strategies must be put in place to limit mortality among T2DM people, especially in developing African countries such as Morocco. Addressing diabetes in Morocco, like in all developing countries in Africa, requires a multifaceted approach involving public health education, improved healthcare access, and community support to encourage healthier lifestyles.

### What is known about this topic

- Type 2 diabetes mellitus is considered to be the most serious endocrine disease in Africa;
- In Africa, this pathology is constantly on the increase and causes many deaths: despite the strategies undertaken, diabetes continues to increase and cause many deaths;
- Morocco, like all developing African countries, is suffering from the spread of type 2 diabetes as a result of lifestyle changes; obesity resulting from overeating favors the onset of diabetes: diabetes self-management is poorly practiced in Morocco.

### What this study adds

- Limiting the complications of type 2 diabetes requires good self-management of endocrine disease;
- Therapeutic education is still strongly recommended for good control and monitoring of diabetes: if T2DM is well managed by the patient, complications can be limited and the patient's life expectancy will be longer;
- Physical activity and regular treatment are the first precautions to be taken in order to limit complications in diabetic subjects.
